# Cutting mechanism of straight-tooth milling process of titanium alloy TC21 based on simulation and experiment

**DOI:** 10.1371/journal.pone.0258403

**Published:** 2021-10-14

**Authors:** Zhang Lei, Lei Pei

**Affiliations:** College of Mechanical and Energy Engineering, NingboTech University, Ningbo, China; University of Vigo, SPAIN

## Abstract

Due to the characteristics of high strength, high chemical activity and low heat conduction, titanium alloy materials are generally difficult to machine. As a typical titanium alloy with higher strength and lower heat conductivity, the machinability of titanium alloy TC21 is very poor and its cutting process is companied with larger cutting force and rapid tool wear. Straight-tooth milling tool is often used to cut the groove and side surface in the titanium alloy parts. And the milling method can be used to investigate the cutting mechanism because the cutting force has only two components and the better chip morphology is obtained. To investigate the straight-tooth milling process of TC21 alloy, a series of milling force experiments have been done. In addition, a 3D finite element model (FEM) for the straight-tooth milling process of TC21 alloy is presented to simulate the milling process. In the model, the constitutive material model, the failure model, the friction model and the heat transfer model were adopted. Through the simulation, chip formation, stress distribution, cutting force and milling temperature were obtained. The cutting force reaches its maximum when the spindle speed reaches about 13000 rpm, and then decreases as the speed continues to increase. The results confirmed that the similar “Salomon” phenomenon existed in the cutting process of TC21 alloy.

## Introduction

Titanium alloy is a versatile metal material, which can be used in aviation, marine, biomedical and sports fields [1, 2]. Titanium alloy generally has high strength, high chemical activity and low thermal conductivity properties, belongs to the difficult to process material, resulting in low cutting efficiency, fast tool wear, poor cutting quality [3, 4].

The research of cutting mechanism is very important for the improvement of cutting technology. Many researchers have done a lot of work to explore the cutting mechanism of various metal materials. Bayraktar [5] investigated the effects of copper and zinc additions on the machinability properties of Al–7Si alloy and the structural and mechanical properties of the alloys with conventional methods. Toubhans [6] evaluated the machinability of Inconel 718 in the turning with a round carbide tool and analyzed the evolution of cutting forces in tool life. Bayraktar [7] measured the mechanical properties of Al-12Si-0.6Mg and performed a series of cutting experiment to observe the cutting force. In the investigation of cutting, cutting force is one of the most important objects. Pimenov [8] established a numerical model of cutting force in orthogonal cutting. Bayraktar [9] investigated the cutting force in milling of a carbon-fiber-reinforced polymer composite material under various cutting tools and parameters. Do [10] presented a cutting force model based on the five-axis milling experiment validation of aerospace parts. Kang [11] developed the improved cutting force model of brittle and hard materials in the ultrasonic assist grinding process.

Up to now, there have been many researches on the cutting mechanism of titanium alloy. Jawaid [12] analyzed the wear mechanism of coated carbide tool when cutting titanium alloy Ti6Al4V under various cutting conditions. Sutter [13] studied the mechanism of chip formation in high speed cutting of titanium alloy with uncoated carbide tools by orthogonal experiment. Xiong [14] studied the cutting mechanism of titanium alloy Ti6Al4V in ultra-precision cutting process. Pimenov [15] discussed various cooling and lubrication method for improving the machinability of ti and its alloys. Lou [16] attempted to use the electro-pulsing treatment method to improve the machinability of titanium alloy in the ultra-precision turning using a single point diamond tool.

The above researches are based on the experimental methods. In recent years, finite element technique has been used more and more to explore the cutting mechanism. Many finite element models have been established to investigate the cutting process of titanium alloys. Thepsonthi [17] simulated the micro-end milling of titanium alloy using an established 3D finite element model. Hu [18] used the finite element technique to study the mechanism of chip formation in the ultrasonic assist ultra-precision cutting of titanium alloy. Wu [19] developed a 3D milling model to simulate the cutting model to investigate the cutting phenomenon of titanium alloy.

Titanium alloy TC21 is a new type of high-strength alloy, its cutting process existed many problems, including rapid tool wear, high cutting temperature and poor cutting quality. There are very few literatures on cutting mechanism of this alloy. Zhang [20] investigated tool wear and cutting force of TC21 alloy in high speed milling with physical vapor deposition coated carbide tools under different cutting conditions. Li [21] used the minimum quantity lubrication method in milling of TC21 with graphene nanofluid for evaluating the influence of graphene nanoparticle on the surface quality. Zhang [22] established an oblique cutting model to analyze the influence of tool parameters on cutting force and residual stress in cutting of TC21 alloy. Sun [23] used the turn-milling method to improve the machinability and optimized the cutting parameters of TC21 alloy in cutting process. These studies have made some attempts on the cutting of titanium alloy TC21, but none of them have systematically studied this material from any aspect.

In order to investigate the cutting mechanism of TC21 alloy, a series of straight-tooth milling experiments have been done. At the same time, the finite element method is used to assist in revealing the straight-tooth milling process of TC21 alloy. In cutting simulation, the material constitutive model, failure model and friction model have been applied. The phenomena of chip generation, cutting force change and cutting temperature change were analyzed through the simulation of straight tooth milling process. In order to study the straight tooth cutting process of titanium alloy, the cutting experiment was carried out. Finally, the relationship between cutting force and cutting speed was studied through a series of cutting force experiments.

## Materials and methods

### Setup of experiment

In this study, the material type of the workpiece is titanium alloy TC21. This alloy is composed of alpha phase and beta phase. The blank of titanium alloy is made by forging under the temperature 989°C. The forging process is divided into two steps, and the deformation is 50% in each step. The material is cooled by air cooling method after forging, and the heat treatment method is 900C/2h, 590°C/2h. The X-ray energy spectrum analyzer STEREOSCAN 600 X is used to get the chemical elements of TC21 alloy and the chemical composition is listed in Table 1. The Rockwell Hardness tester HD-1875 is used to obtain the surface hardness and five points have been tested. The mechanical properties of titanium alloy TC21 after forging and heat treatment are very superior. The physical properties of TC21 alloy are listed in Table 2.

**Table 1:** Chemical elements of TC21 alloy.

Al	Mo	Nb	Sn	Zr	Cr	Ti
5.8	2.4	1.8	2.3	1.9	1.2	Others

**Table 2:** Physical properties of TC21 alloy.

Properties	Values
Density(kg/m^3^), *ρ*	4610
Yield strength (MPa),*σ*_*s*_	1280
Hardness (HRC)	42
Elastic modulus (GPa),*E*	163
Thermal conductivity(W/mk),*λ*	6.3
Poisson’s ratio,*μ*	0.31

**Fig 1:**
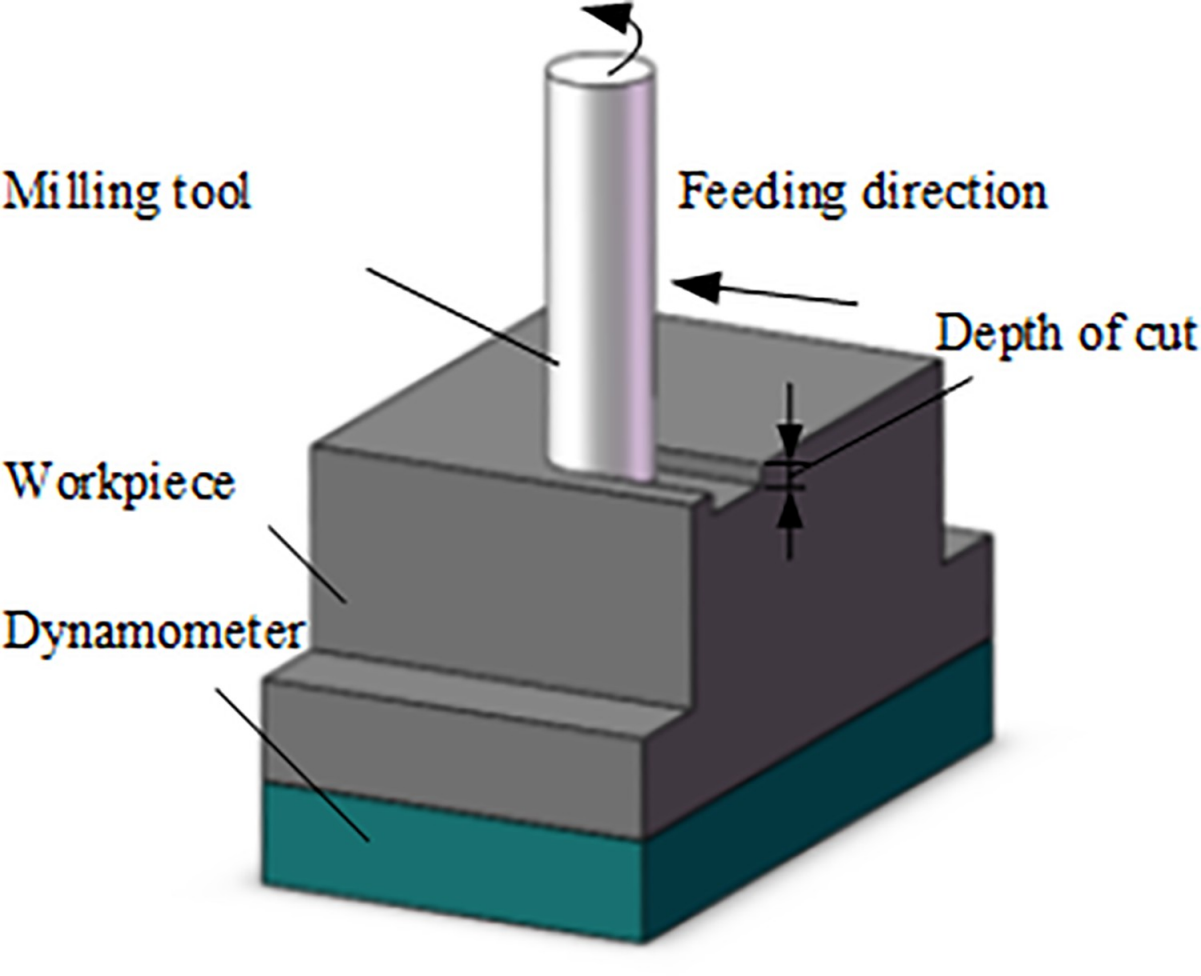
Schematic of straight-tooth milling experiment.

**Fig 2:**
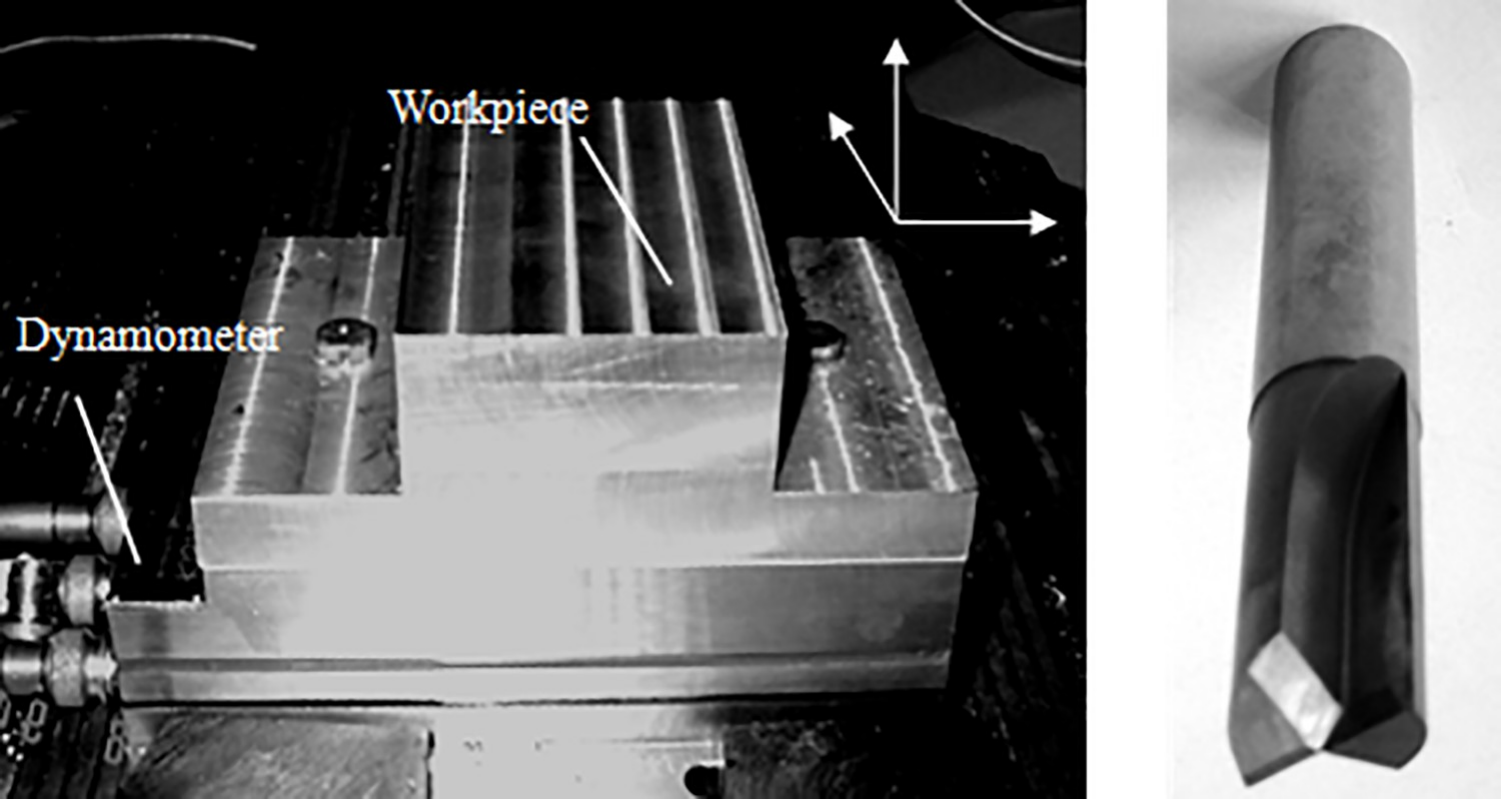
Experiment setup of the straight-tooth milling. (a) Sample of TC21, (b) Straight-tooth milling tool.

In this paper, a series of straight-tooth milling experiments of TC21 alloy have been done on a CNC machine Fidia HS664RT. The 2 teeth straight-tooth milling tool was used in the milling tests. The material of the milling tool is TiAlN coated carbide. The tool rake angle is 10° and the tool clearance angle is 15°.The schematic of straight-tooth milling experiments is shown in Fig 1. The practical setup of cutting force experiments is shown in Fig 2. The Kistler 9257B dynamometer was employed to obtain the milling forces. The detailed milling conditions of titanium alloy TC21 are listed in Table 3.

**Table 3:** Straight-tooth milling conditions of TC21 alloy.

Parameters	Values
Milling depth (mm)	1
Spindle speed (rpm)	2000–18000
Feed per tooth (mm)	0.05,0.1,0.15
Diameter of milling tools (mm)	10,15,20
Number of teeth	two
Rake angle	10°
Clearance angle	15°
Cutting environment	Dry cutting

### 3D finite element model of TC21 alloy

#### Setup of finite element model

**Fig 3:**
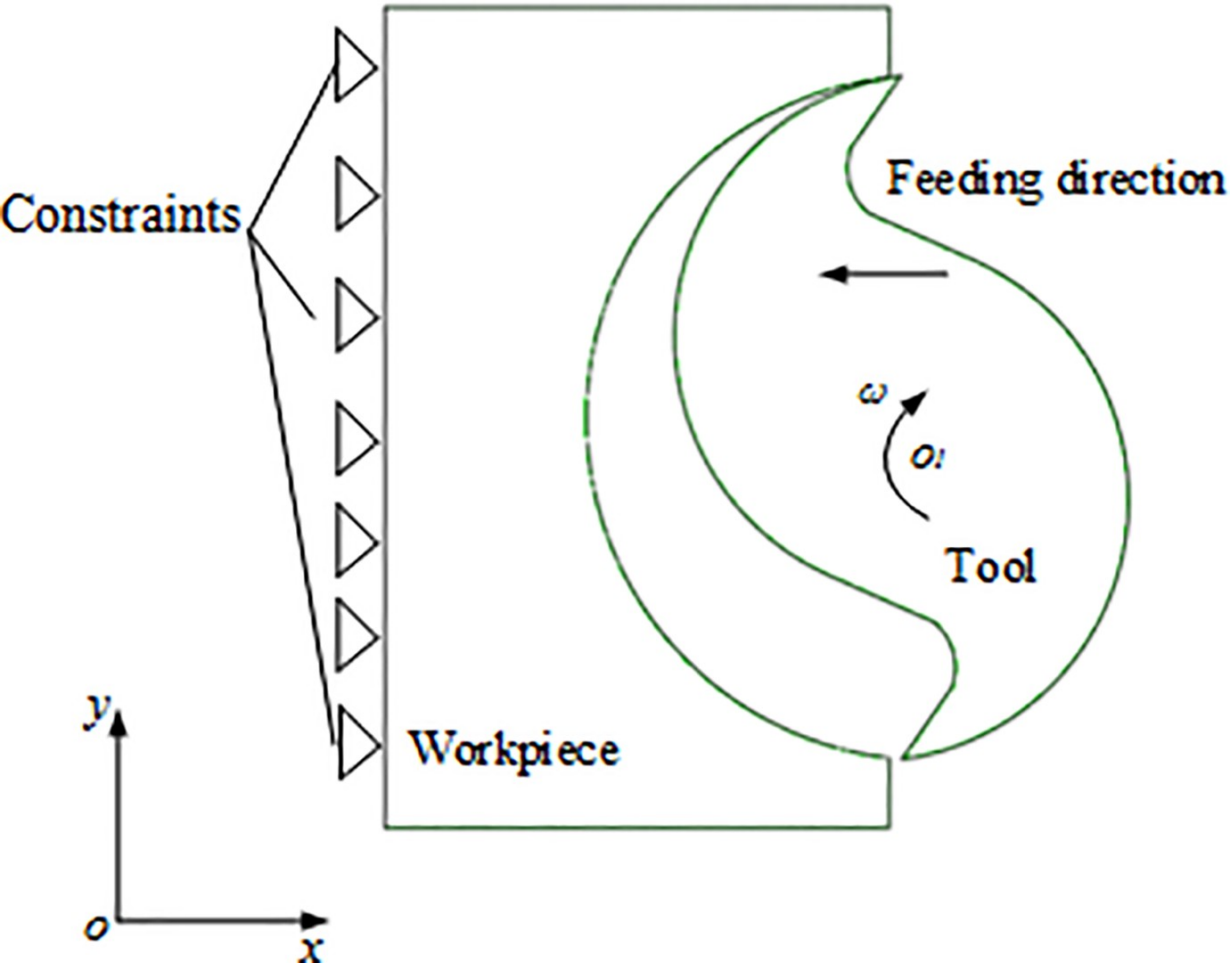
Schematic of cutting process.

**Fig 4:**
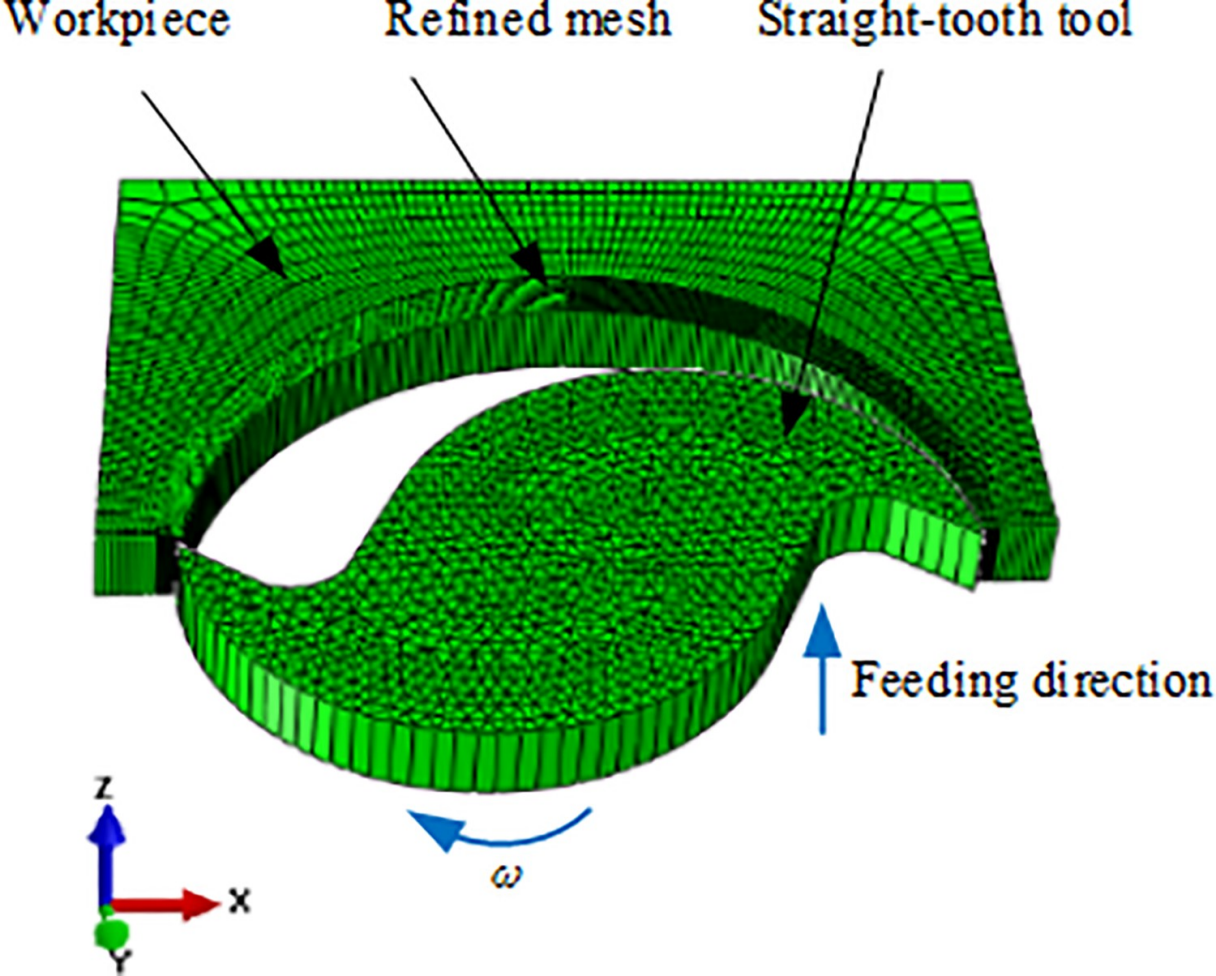
Cutting model for straight-tooth milling of TC21 alloy.

Metal cutting is a complex process of thermal, mechanical and chemical interactions. In recent years, finite element method has been widely used in the research of cutting process because it can reduce the cost of cutting experiment and obtain cutting data which is difficult to obtain in the cutting experiment. A 3D finite element model for the straight-tooth milling process of titanium alloy TC21 has been established using the finite element software ABAQUS. The schematic of straight-teeth milling process is shown in Fig 3. The initial mesh and configuration of the workpiece and tool are shown in Fig 4. In cutting simulation, the mesh must be sufficiently refined to form the accurate chip morphology. To improve the efficiency and precision, the mesh in the cutting region has been refined, while the residual mesh was set to be sparse. The numerical model is made of 121,600 hex elements, and the element type is C3D8RT. The element is an 8-node thermally coupled brick, trilinear displacement and temperature, reduced integration, hourglass control and the type of element is easy to get the solution. The height of workpiece is 2 mm. The milling environment is dry cutting. The initial temperature is set to be 20°C. The tool is defined as a rigid-body and has 5,156 elements. The milling tool has two teeth. The tool diameter is 20 mm, tool rake angle is 10° and the clearance angle is 15°.The simulation took about 200 hours using the computer with one Xeon W-2123 CPU and 64GB Ram.

#### Material model

The Johnson-Cook plasticity model is a particular type of Mises plasticity model with analytical forms of the hardening law and rate dependence [24]. The model is suitable for high-strain-rate deformation of many materials, including most metals and typically used in adiabatic transient dynamic simulations Johnson-Cook hardening is a particular type of isotropic hardening where the static yield stress. The yield stress is expressed as

$$\sigma =(A+B\varepsilon^{n})(1+C\;\ln\frac{\dot{\varepsilon }}{{\dot{\varepsilon }}_{0}})[1-{\left(\frac{T-T_{r}}{T_{m}-T_{r}}\right)}^{m}]$$ (1)

Where *ε* is equivalent plastic strain, $\dot{\varepsilon }$ is equivalent plastic strain-rate, ${\dot{\varepsilon }}_{0}$ is reference strain-rate, *T*, *Tm* and *Tr* are material, melting and room temperature, respectively. *A*, *B*, *C*, *n* and *m* are the J-C constants. The J-C model data of TC21 alloy in simulation are come from the literature [25] and listed in Table 4.

**Table 4:** Constants of Johnson–cook equation for TC21 alloy.

Parameters	Values
*A* (MPa)	1150
*B* (MPa)	870
*n*	0.35
*C*	0.22
*M*	2.15
*T*_*r*_ (°C)	20
*T*_*m*_ (°C)	1758

#### Failure model

A dynamic failure model of Johnson-Cook plasticity model was used in the simulation [26]. This model is suitable only for high-strain-rate deformation of metals. The Johnson-Cook dynamic failure model is based on the value of the equivalent plastic strain at element integration points, failure is assumed to occur when the damage parameter exceeds 1. The failure model can be expressed by:

$$\dot{\varepsilon_{f}}=[D_{1}+D_{2}\;\exp(D_{3}\frac{p}{q})][1+D_{4}\;\ln(\frac{\dot{\varepsilon }}{\dot{\varepsilon_{0}}})](1+D_{5})$$ (2)

Where *D*_*1*_–*D*_*5*_ are the damage constants, respectively. *p* is the hydrostatic pressure, *q* is the Mises stress, $\dot{\varepsilon_{0}}$ is the reference strain rate, $\dot{\varepsilon }$ is the strain at the time of failure. The material failure parameters of TC21 alloy come from the literature [23] and listed in Table 5.

**Table 5:** Fracture parameters of Johnson–cook for TC21 alloy.

Parameters	Values
*D*_*1*_	0.04
*D*_2_	0.52
*D*_3_	0.3
*D*_4_	0.05
*D*_5_	2.7

#### Frictional model

Contact action has very significant influence on the metal cutting process. Friction between the contact interfaces of tool and chip will result in tool wear because of high temperature, high stress and chemical reaction. There are two zones in the contact area of chip and tool, the sliding and sticking zone [27]. In the sliding zone, friction needs to abide by the Coulomb law. But in the sticking zone, the shear stress must be equal to the critical friction stress. This contacted model between chip and tool is expressed by the following equation:

$$\tau_{f}=\tau_{s},\mu \sigma_{n}\geq \tau_{s}\;(Sticking\;zone)$$ (3)

$$\tau_{f}=\mu \sigma_{n},\;\mu \sigma_{n}<\tau_{s}\;(Sliding\;zone)$$ (4)

Where *τf* is friction force and *μ* is friction coefficient between workpiece and tool. *σ*_*n*_ is normal stress on contact surface, *τ*_*s*_ is ultimate shear flow stress. By combining the model with the material, the friction status between the chip and rake face can be well reflected. In the cutting simulation, the frictional state will be decided automatically based on the value of contact stress. In the cutting simulation of TC21 alloy, the revised Coulomb’s law is used to represent the contact action between chip and rake face. In the simulation, the frictional coefficient is set to be 0.31.

## Results and discussion

**Fig 5:**
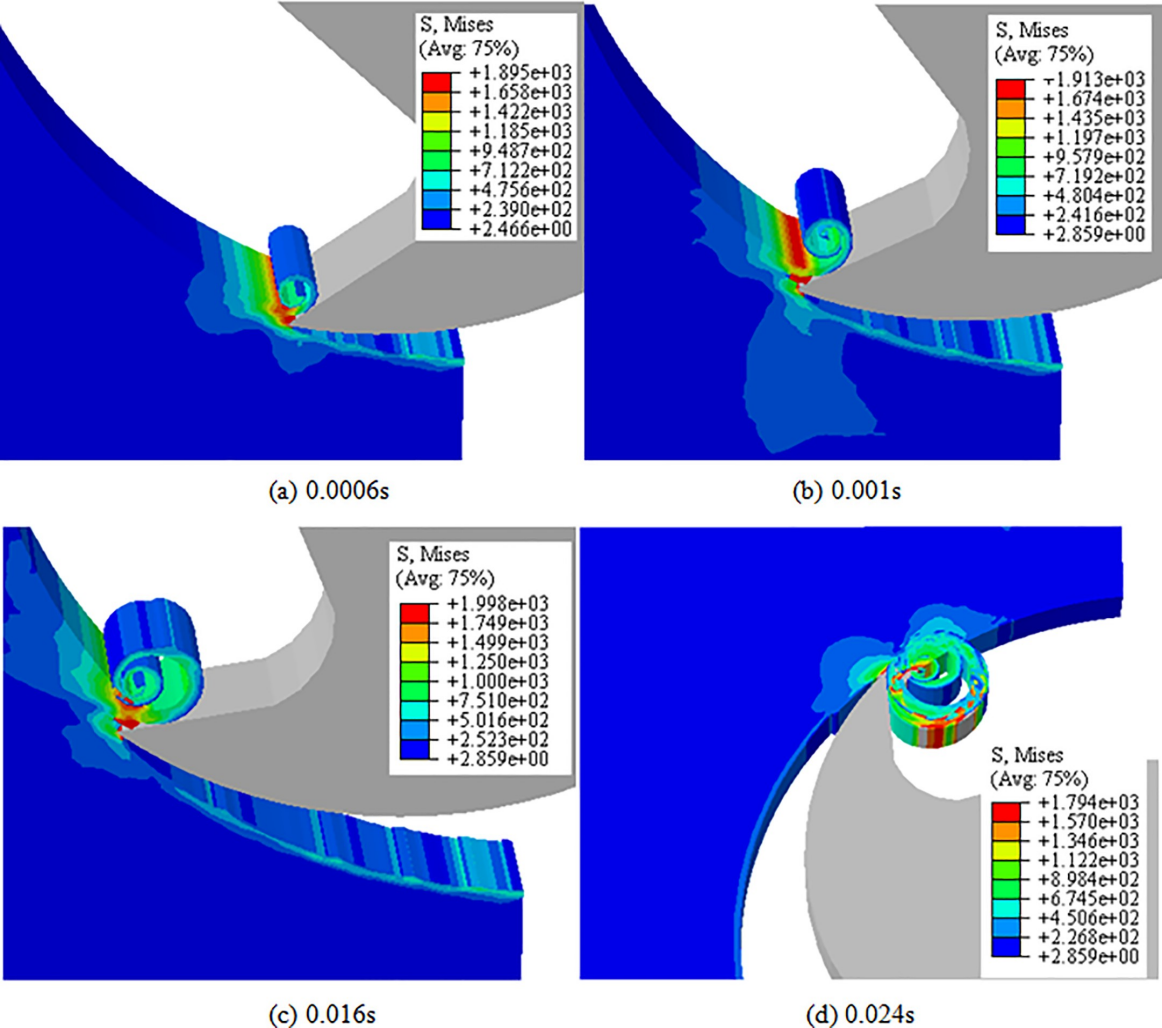
Chip formation and stress distribution at different cutting times. (a) 0.0006s, (b) 0.001s, (c) 0.016s, (d) 0.024s. (Spindle speed: 8000 r/min, and feed per tooth: 0.1 mm).

The cutting process of titanium alloy TC21 was studied by a series of cutting experiments and numerical simulation. The 3D finite element model for the straight-tooth milling process of TC21 was developed using the software ABAQUS 6.16. In the simulation of the milling process, the analysis step “Dynamic, temp-disp, explicit” was selected. The chip formation and stress distribution in the simulation of the milling process for TC21 alloy at different cutting times are shown in Fig 5. The chip separated and curled with the cutting of tool. The maximum Mises stress occurred in the primary shear band and the value reached 1998MPa.

**Fig 6:**
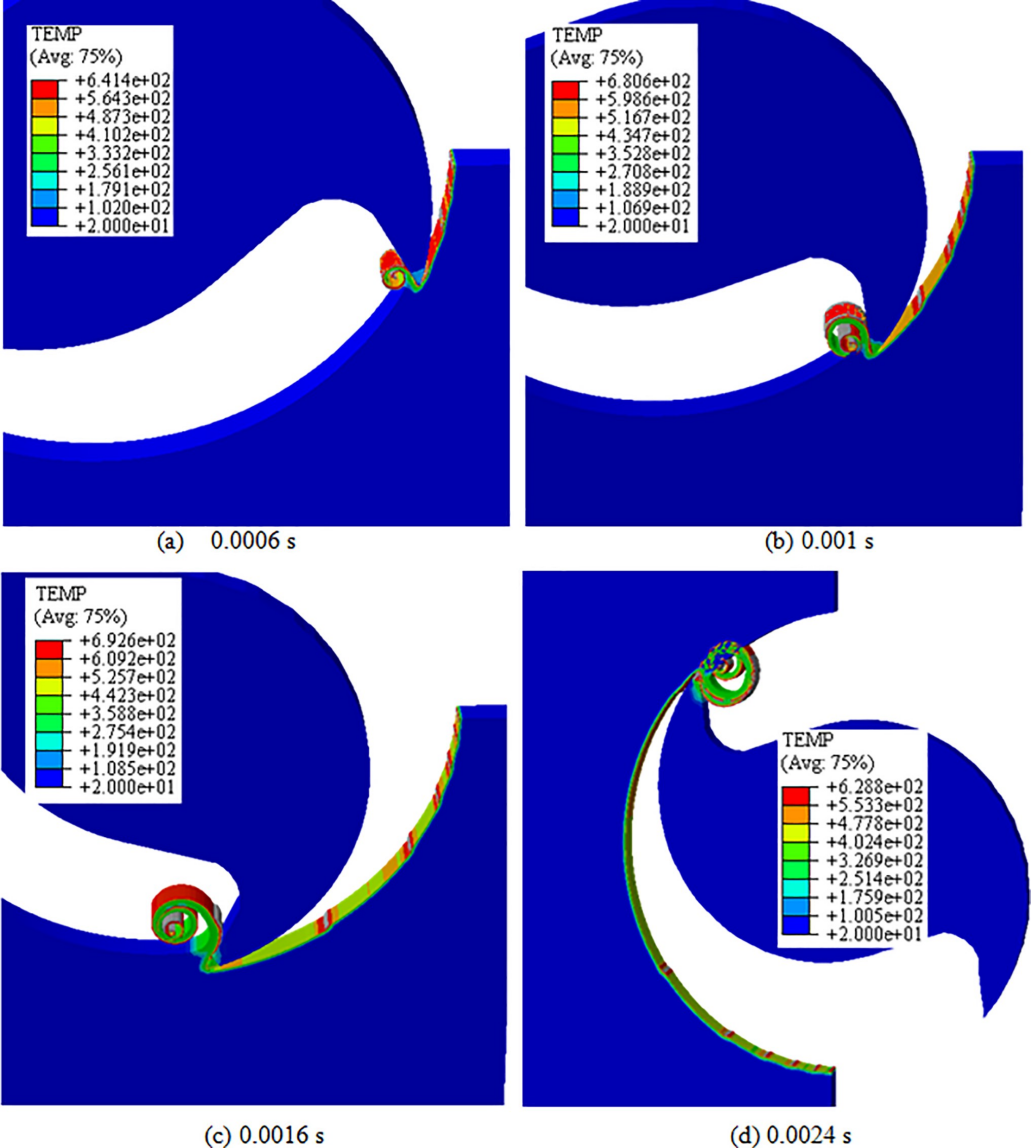
Temperature distribution at different cutting times. (a) 0.0006 s, (b) 0.001 s, (c) 0.0016 s, (d) 0.0024 s. (Spindle speed: 8000 r/min, feed per tooth: 0.1 mm).

The temperature distribution obtained by simulation in the milling process of alloy TC21 during the different cutting times is shown in Fig 6. It shows that the maximum temperature is 692.6°C when the tool cuts into the middle of workpiece. And the highest temperature occurs in the main deformation zone in the workpiece, not on the contact surface between the tool and the chip. Because the heat conductivity of titanium alloy TC21 is very low, the cutting process can be seen as an adiabatic state. In the cutting, the heat of deformation energy cannot be quickly transmitted to the tool when the cutting speed is high, so the tool temperature is lower than the temperature at the shear zone of workpiece. Adiabatic effect is also thought to be responsible for the formation of serrated chip.

**Fig 7:**
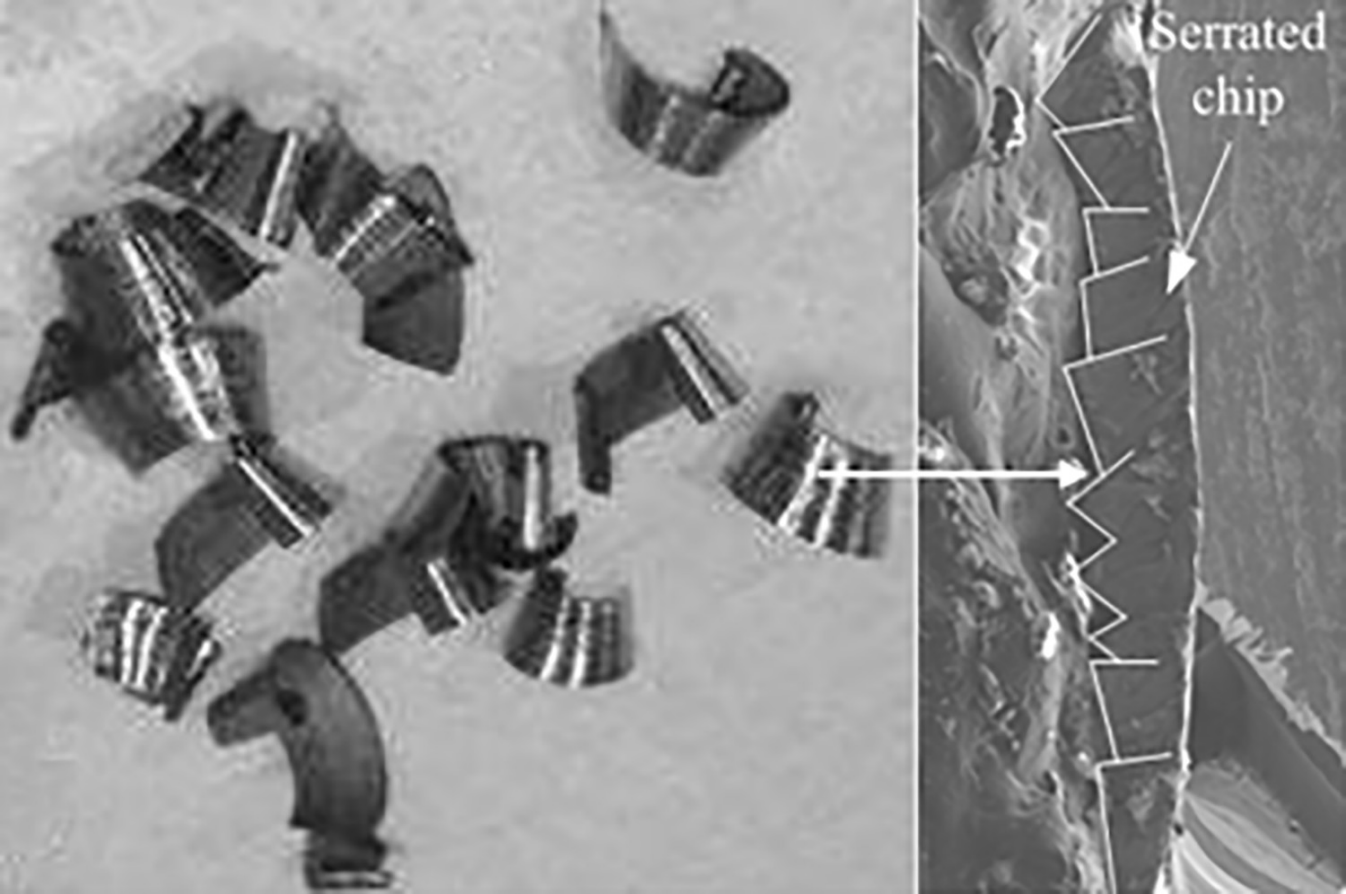
Chip shape of titanium alloy in strait-tooth milling. (Spindle speed: 8000 r/min, and feed per tooth: 0.1 mm).

Fig 7 is the chips in the straight-tooth milling experiments of titanium alloy TC21. It can be seen that the shape of chips is serrated. In addition, the experimental results showed that the chip of titanium alloy TC21 is serrated in the range of the experimental cutting parameters. The serrated chip shape proved that the titanium alloy TC21 has a strong adiabatic effect. The formation of the serrated chip in the milling process will lead to the fluctuation of the milling force. Cutting force fluctuations can cause the vibration of tool and machine system, increase tool wear and reduce the quality of machined surfaces.

**Fig 8:**
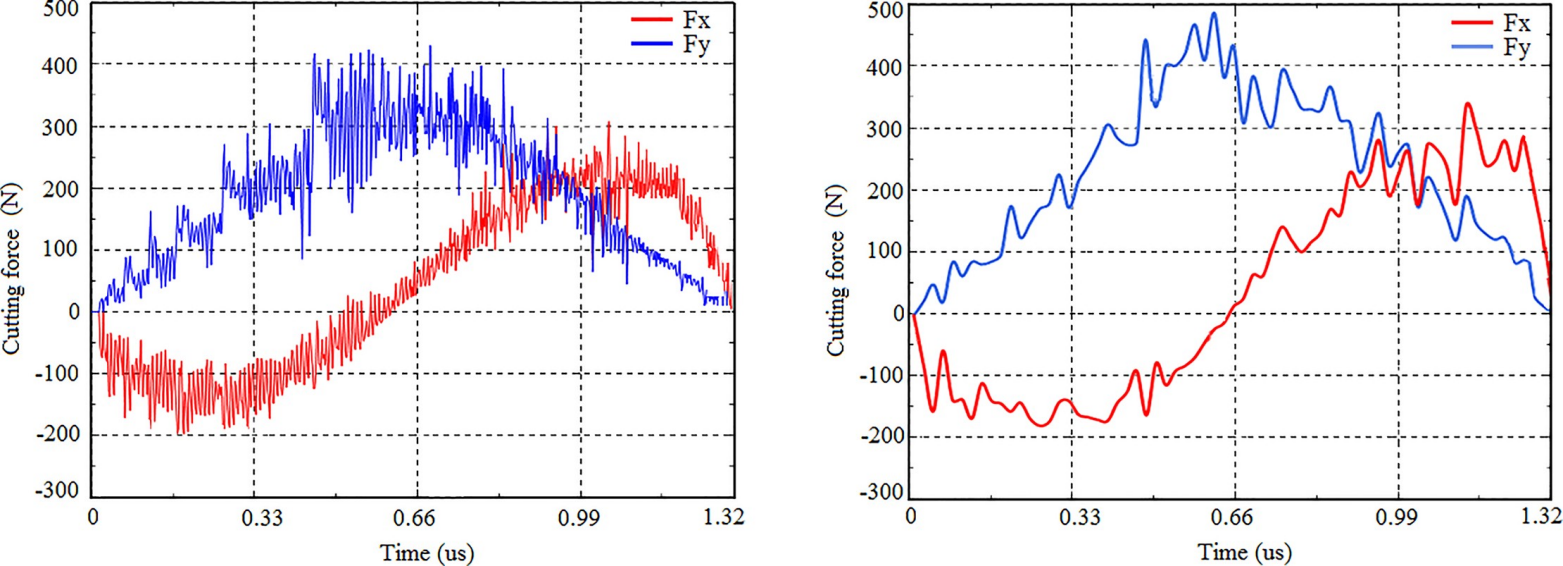
Milling forces curves of titanium alloy TC21. (a) Milling force curve in simulation, (b) Milling force curve in experiment.

Fig 8A is the milling force curves obtained by the developed 3D FEM simulation, and Fig 8B is the milling force curves measured by the milling experiment at the same milling conditions (spindle speed: 8000 r/min, and feed per tooth: 0.1 mm). Because the chip thickness is constantly changing, the cutting force also presents periodic changes. The maximum values of cutting forces in two directions during cutting experiment and cutting simulation are compared. The error of milling forces between experiment and simulation is less than 9.5%. It shows a good agreement of milling forces between the experiment and the simulation.

**Fig 9:**
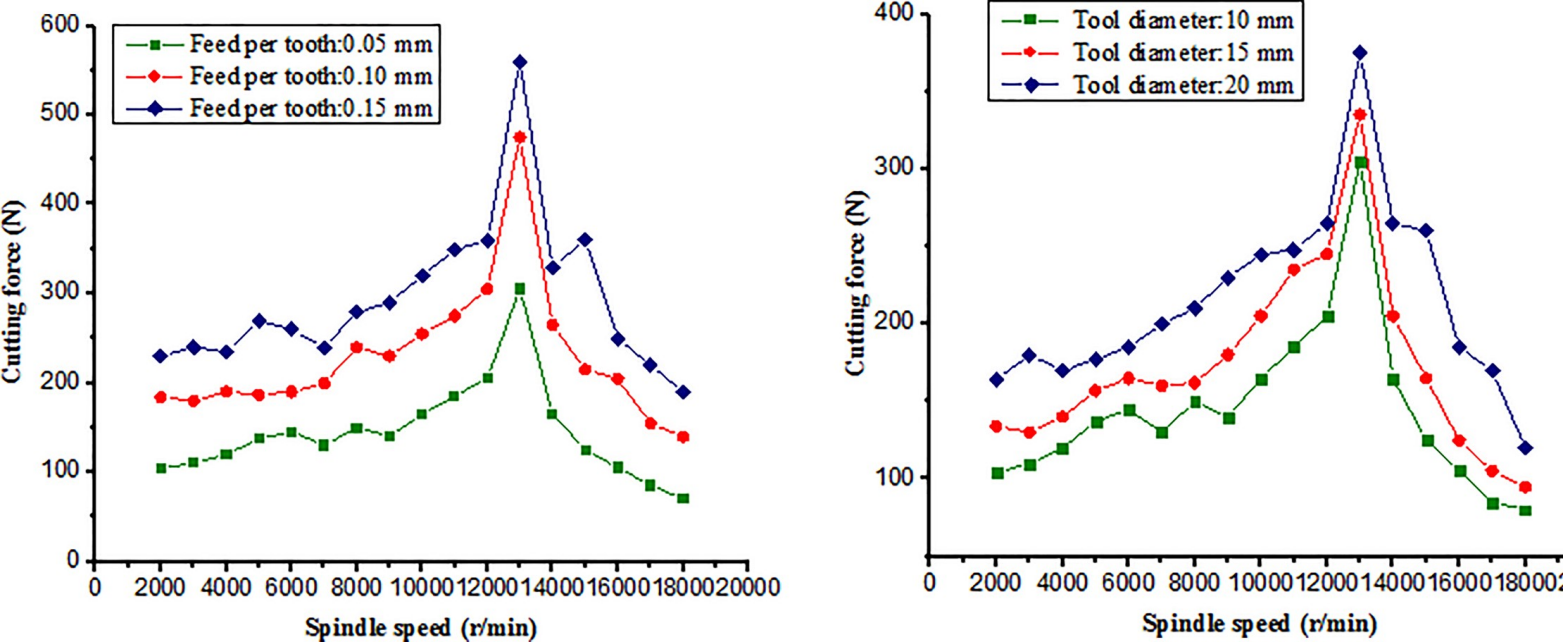
Comparison of milling forces. (a) Milling force with the different feed per tooth (Tool diameter: 10 mm), (b) Milling force with the different tool diameters (Feed per tooth: 0.05mm).

Fig 9 shows the influence of different spindle speeds on cutting force “Fy” under the different feed per tooth and different diameters of milling tool. It can be seen that the similar “Salomon” phenomenon is very obvious. As the spindle speed increases, the cutting force increases. To a critical cutting speed, the cutting force increases significantly, and then the cutting force decreases significantly as the cutting speed increases. The results proved that the high cutting speed above the critical speed can reduce the cutting force and improve the cutting efficiency of titanium alloy TC21.

## Conclusions

In this study, aiming at the difficult machinability of titanium alloy TC21, a series of straight-tooth milling force experiments have been done. In addition, a 3D finite element model (FEM) for the straight-tooth milling process of titanium alloy TC21 is proposed to study its cutting mechanism. In the model, the constitutive material model, the failure model and the friction model were adopted. Through the simulation, chip formation, stress distribution, cutting force and milling temperature were obtained. The conclusive findings are as follows:

1. The main cutting force reaches its maximum when the spindle speed reaches about 13000 rpm, and then decreases as the speed continues to increase. The results confirmed that the similar “Salomon” phenomenon existed in the cutting process of TC21 alloy based on the cutting force experiments.
2. The results of cutting force proved that the high speed cutting can reduce the cutting force and improve the cutting efficiency of titanium alloy TC21.
3. Through the comparison of cutting force, the error of cutting force is less 9.5% and the finite element model have been verified.
